# Determination of 4-Hexylresorcinol in Shrimp Samples by Solid Phase Extraction Ultra Performance Liquid Chromatography-Tandem Mass Spectrometry

**DOI:** 10.3390/molecules23092173

**Published:** 2018-08-29

**Authors:** Yunbin Hao, Xuehui Gao, Wenshui Xia

**Affiliations:** 1 State Key Laboratory of Food Science and Technology, School of Food Science and Technology, Collaborative Innovation Center of Food Safety and Quality Control in Jiangsu Province, Jiangnan University, Lihu Avenue 1800, Wuxi 214122, Jiangsu, China; yunbinhao@163.com; 2 School of Fishery, Zhejiang Ocean University, Haida Road 1, Zhoushan 316021, Zhejiang, China; 15858083495@163.com

**Keywords:** solid phase extraction (SPE), ultra performance liquid chromatography-tandem mass spectrometry (UPLC-MS/MS), shrimp samples, 4-Hexylresorcinol (4-HR)

## Abstract

A method for the rapid determination of 4-hexylresorcinol (4-HR) residue in shrimp by solid phase extraction (SPE) ultra-high performance liquid chromatography-tandem mass spectrometry (UPLC-MS/MS) was established. 4-HR was extracted twice with methanol, and the extract was formulated into methanol-water solution (1:1). After being cleaned up and concentrated by a PRIME HLB solid phase extraction column, the sample was analyzed by UPLC-MS/MS and quantitatively determined by an external standard method. The separation was performed with a gradient system consisting of water and acetonitrile as the mobile phase. Monitoring was performed by electrospray ionization (ESI) in negative ion mode using multiple ion reaction monitoring (MRM). Good linearity was obtained in the concentration range of 1.0–100.0 μg/L, with correlation coefficients larger than 0.999. The limit of detection (LOD) was 0.25 μg/kg and the limit of quantification (LOQ) was 0.80 μg/kg. The average recoveries of 4-HR at spiked concentrations of 2.40, 6.40 and 16 μg/kg ranged from 81.35% to 94.68% with the relative standard deviations (*n* = 6) from 3.57% to 6.86%. The results showed that the method is simple, fast, sensitive, reliable, and reproducible; thus, it could be used as a rapid confirmation and quantitative analysis method of 4-HR residue in aquatic products.

## 1. Introduction

**Figure 1:**
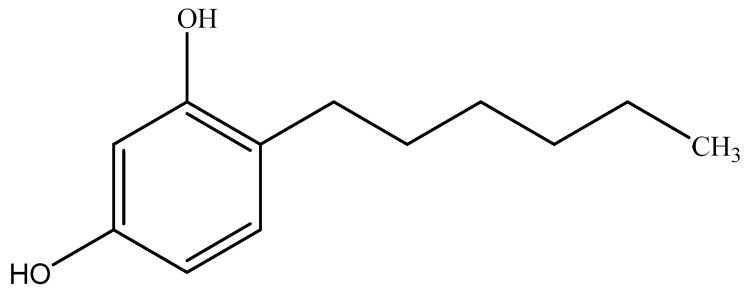
Structure of 4-hexylresorcinol.

As a nutrient-rich aquatic product, shrimp is a popular food, and its quality concerns consumers. Shrimp is a perishable product, and melanosis affects its appearance, degrades its quality, and reduces its value [1,2,3,4]. Melanosis is initially caused by the biochemical action of polyphenoloxidase, which oxidizes phenols to quinines. Subsequently, nonenzymatic polymerization of the colourless quinones gives rise to black, high-molecular-weight pigments, and very dark or black coloring [5,6]. Aquatic preservatives are used in the market to extend shelf life and freshness. 4-Hexylresorcinol (4-HR, molecular formula: C_12_H_18_O_2_, Figure 1) is a novel anti-oxidant, a preservative, and a blackening inhibitor used in shrimp products. In 1991, Mcevily [7,8,9] first proposed that 4-HR is an effective inhibitor of phenoloxidase in shrimp, which can safely replace sodium metabisulfite to inhibit the browning of the shrimp body. Subsequently, it was found that, as the component of preservatives, 4-HR could also inhibit the browning of apples [10], Pleurotus eryngii [11], and Coprinus comatus [12]; it also has many applications in aquatic products. The increased concentration of 4-HR and its residue has become another important safety issue [13,14]. China′s "Sanitation Standards for the Use of Food Additives” (GB2760-2014) stipulates that the maximum usage in fresh aquatic products (shrimp only) is ≤1 mg/kg, and it can be used in moderation according to the needs of production. To meet the requirements of preservation and color protection, the added amount of 4-HR in the market often exceeds the standard, and it cannot be completely removed when ingested, thus causing some harm to the human body [15]. Considering that the use of food additives is inevitable, it is necessary to establish a sound detection method to strengthen the monitoring of 4-HR.

At present, gas chromatography-mass spectrometry [16], high performance liquid chromatography [17,18,19,20,21], and ultra-high performance liquid chromatography-tandem mass spectrometry methods [22] are mainly used to detect 4-HR in aquatic products in China and across the world. High performance liquid chromatography-tandem mass spectrometry (LC-MS/MS) has been increasingly used due to its advantages such as high selectivity, high sensitivity, and high throughput [23]. Mass spectrometry can detect 4-HR with relatively high sensitivity and accuracy, and can be used to determine the molecular weight and chemical structure. Ultra-high performance liquid chromatography provides good separation. The combination of this technique with mass spectrometry can be used to accurately analyze the compounds qualitatively and quantitatively. This method has become an increasingly important technology in the application of drug residue detection in food [24,25]. According to the chemical structure and properties of 4-HR, a C18 solid-phase extraction column is often used, but purification is not ideal. In this study, a PRIME HLB solid phase extraction column was used for purification, which effectively reduced the impact of matrix effects. PRIME HLB is a versatile solid phase extraction column that can be used for the analysis of a wide range of drug residues in aquatic and animal-derived samples; however, its application in aquatic products is rarely reported. In this experiment, a PRIME HLB solid-phase extraction column was used for purification, and combined with the high sensitivity and accuracy of ultra-high performance liquid chromatography-tandem mass spectrometry, a set of methods for 4-HR detection in aquatic products with high efficiency, speed, and sensitivity was established. The method has the advantages of reduced matrix interference, strong specificity, good repeatability, and good recovery, and it can be applied to the qualitative analysis and quantitative detection of 4-HR residues in real samples.

## 2. Results and Discussion

### 2.1. Optimization of the Chromatography Conditions

The common C18 column analysis of 4-HR failed to produce good peak shapes. In this study, an ACQUITY UPLC BEH C18 column with a particle size of 1.7 μm was used to analyze the target compound. When the ammonium acetate solution was used as the mobile phase, there were many interference peaks from impurities, and the response value was decreased and residuals were observed. The response value of the standard solution could not be accurately determined. Therefore, water was selected as the mobile phase. Under optimized chromatographic conditions, the peak shape of 4-HR was sharper, and the target compound separation and column equilibration could be completed within about 5.0 min. Using the 10.0 μg/L 4-HR standard solution, we compared the separation ability of the mobile phase, either with aqueous methanol or acetonitrile as the organic phase. When acetonitrile was used as the organic phase, the 4-HR sensitivity was slightly higher than when methanol was used as the organic phase, and the peak time was about 2 min with a sharp peak. In contrast, when methanol was used as the organic phase, the peak time was later, which extended the analysis time. Based on these data, acetonitrile-water was chosen as the mobile phase.

### 2.2. Optimization of the MS Condition

**Figure 2:**
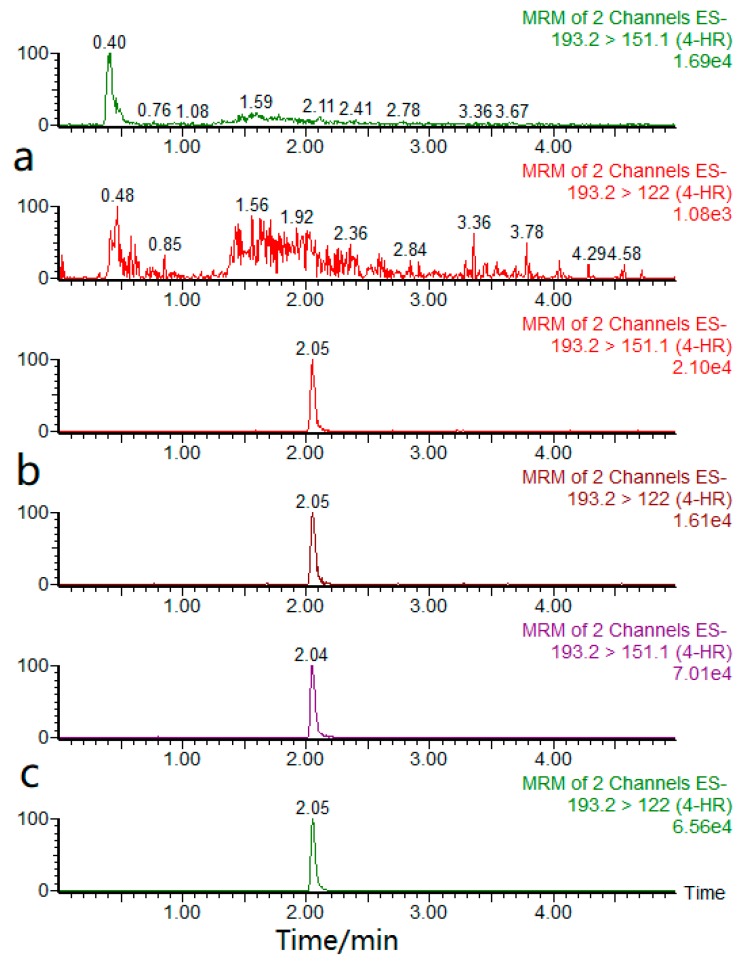
MRM chromatograms of 4-HR (**a**) blank sample, (**b**) shrimp spiked sample and (**c**) standard solution.

4-HR is a weakly acidic substance and was usually analyzed by ion spray scanning. Under the negative ion scanning mode, the optimal MRM MS parameters of the target compound were tuned using a 0.2 μg/mL 4-HR standard solution. The 4-HR molecular ion was determined by first-order MS full scan analysis. The most suitable cone voltage and capillary voltage were selected by tuning. [M + H]^+^ (*m*/*z* 193.2) showed the highest abundance, so *m*/*z* 193.2 was selected as the parent ion. The secondary mass spectrometry analysis of the analyte was then conducted to obtain the fragment ion information, and the collision energy and other mass spectrometry parameters were optimized. Finally, the strongest ion (*m*/*z* 151.1) and the second strongest ion (*m*/*z* 122.0) were selected as the qualitative ion and the quantitative ion, respectively. The optimized UPLC-MS/MS ion current graph of 4-HR is shown in Figure 2.

### 2.3. Selection of the Solid Phase Extraction Column

**Figure 3:**
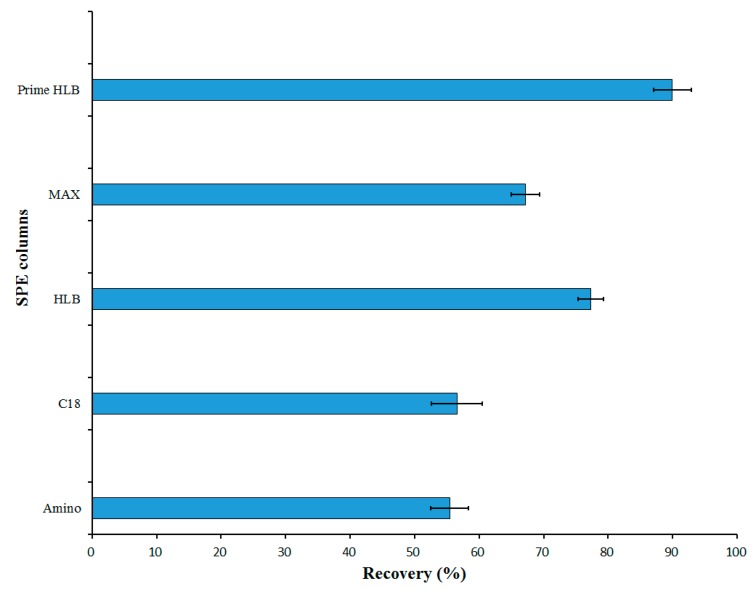
Recovery of different solid phase extraction columns.

In this study, the following columns were used for solid phase extraction: C18, HLB, MAX, amino, and Prime HLB. The best purification column was found by comparing the purification results of the solid phase extraction columns and the recovery rates of 4-HR. Figure 3 displays the recovery rates of different solid phase extraction columns. It can be seen from Figure 3 that all five solid-phase extraction columns could remove the impurities in the sample well. The MAX column and the amino column could only adsorb part of the target, resulting in a low recovery rate. The Prime HLB column had the best adsorption effect and the highest recovery rate. Therefore, the Prime HLB solid phase extraction column was selected for use in the experiment.

### 2.4. Optimization of the Prime HLB Solid Phase Extraction Column Conditions

#### 2.4.1. Selection of the Type and Volume of the Washing Solution

The Prime HLB column was activated with 5 mL methanol and 5 mL water, and the 4-HR spike recovery was increased by about 10%, shortening the experiment time. In this experiment, the effects of different proportions and volumes of washing solutions were compared. When 5% aqueous methanol solution, 10% aqueous methanol solution, 20% aqueous methanol solution, 30% aqueous methanol solution, and 50% aqueous methanol solution were used as the washing solutions, there was no significant difference in effectiveness. However, considering that the sample solution was 50% aqueous methanol, and 4-HR is easily soluble in organic solvent, the washing solution with the higher proportion of organic phase could easily wash off the enriched 4-HR; therefore, 20% aqueous methanol was selected as the washing solution. At the same time, we compared the effect of washing with 3.0 mL, 4.0 mL, 5.0 mL, and 6.0 mL of 5% aqueous methanol. The washing solution with 3.0 mL methanol resulted in a reduced volume and the impurities were not completely washed off. This was especially evident for shrimp samples with more pigmentation, the pigment deposition could clearly be seen in the column. When 6.0 mL washing solution was used, some of the liposoluble impurities could be washed off, which reduced the interference from the shrimp sample matrix and benefitted target elution. In the experiment, 6.0 Ml of 20% aqueous methanol was finally selected to rinse the solid phase extraction column.

#### 2.4.2. Selection of the Eluent

**Figure 4:**
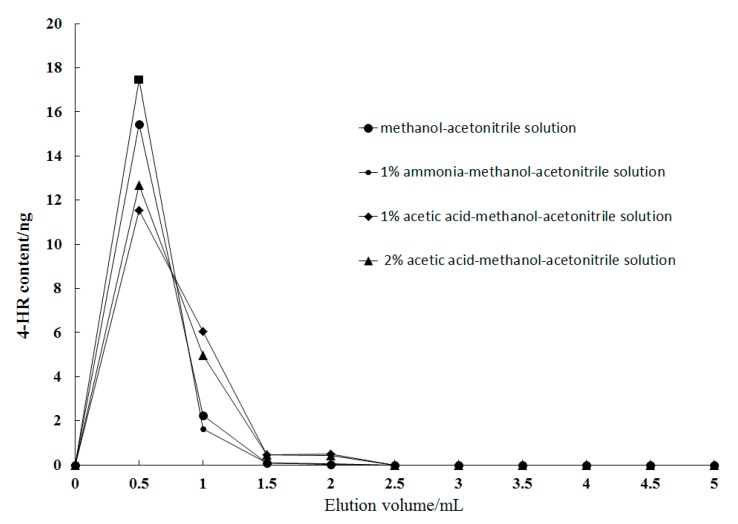
Effect of different elution solvents and elution volumes on the elution efficiency of 4-HR.

The effect of eluents with different pH values, including methanol acetonitrile solution, 1% ammonia-methanol-acetonitrile solution, 1% acetic acid-methanol-acetonitrile solution, and 2% acetic acid-methanol-acetonitrile solution were compared and analyzed. A 20.0 ng 4-HR standard solution was added to 4 centrifuge tubes containing the same amount of 50% aqueous methanol, respectively. After loading and rinsing, 5.0 mL of the 4 eluents were continuously added to the solid-phase extraction column for elution respectively, and every 0.5 mL of effluent was collected in each centrifuge tube. After the collection was completed, the effluents were blow-dried with nitrogen. The mobile phase solution was used to dissolve the effluent and the samples were loaded onto the machine to determine the 4-HR content. The elution curves of 4-HR using the four eluents are shown in Figure 4. The results showed that there were no significant differences in the elution recovery rates of 4-HR among the four elution solutions, among which the elution rate of 2% acetic acid-methanol-acetonitrile solution was faster than that of 1% acetic acid-methanol-acetonitrile solution; the elution rate of 1% ammonia-methanol-acetonitrile solution was the same as that of 2% acetic acid-methanol-acetonitrile solution; 4-HR was not detected in the 4 mL eluent. Considering that an acidic mobile phase could decrease the response value, and in order to ensure high response intensity, 1% ammonia-methanol acetonitrile solution was selected to elute the target in this experiment. The eluent collection volume was selected as 4 mL to complete the collection of the target.

### 2.5. Linear Range and Detection Limit

A 4-HR standard solution was diluted to prepare the following standard working mobile phase solutions: 1.0 μg/L, 5.0 μg/L, 10.0 μg/L, 20.0 μg/L, 50.0 μg/L, and 100.0 μg/L. The standard curve was drawn by using the quantitative characteristic ion mass chromatographic peak area as the vertical axis and the mass concentration of 4-HR standard solution as the horizontal axis. The regression equation was y = 322.552x − 96.7217 in the mass concentration range of 1.0–100.0 μg/L with a good linear range and correlation coefficients all greater than 0.9993. In this method, the detection limit was determined as 0.25 μg/kg by using the 3-fold signal-to-noise ratio and the quantitation limit was determined as 0.80 μg/kg by using the 10-fold signal-to-noise ratio. The quantitation limit of 4-HR in this method was much lower than that determined by liquid chromatography-mass spectrometry method in most other reports [23], and also much lower than the maximum amount (1 mg/kg) stipulated in the “Sanitation Standard for Food Additives” (GB2760-2014) for freshwater aquatic products (shrimp only).

### 2.6. Recovery Rate and Accuracy

Samples from different shrimp species were tested and found to contain no 4-HR. These were weighed accurately and three different concentrations of 4-HR standard solutions of 0.8, 4.0, and 8.0 μg/kg were added. Each addition level was measured 6 times in parallel according to the method described here for 5 consecutive days to calculate the spike recovery rate, the intraday precision and the interday precision. As shown in Table 1, the average recoveries of 4-HR at the three standard concentrations and the five substrates were 81.35–94.68% with relative standard deviations of 3.57–6.86%.

### 2.7. Practical Sample Analysis

To investigate the practicability and applicability of this method, the residual amount of 4-HR in 60 shrimp samples of 5 species from the market, including Parapenaeopsis hardwichii, Fenneropenaeus chinensis, Palaemon gravieri, Acetes chinensis, and Exopalaemon annadalei, were monitored using this method. 4-HR was only detected in 1 shrimp sample, but the amount (16.26 μg/kg) was lower than the maximum stipulated by the national standard. 4-HR was not detected in the rest of the samples. The results of our analysis showed that the method had high recovery rate, its precision and sensitivity could meet the requirements for 4-HR detection, and it can be used in actual sample analysis and determination.

## 3. Materials and Methods

### 3.1. Materials and Reagents

The shrimp samples used in the experiment were Parapenaeopsis hardwichii, Fenneropenaeus chinensis, Palaemon gravieri, Acetes chinensis, and Exopalaemon annadalei, which were all purchased at Dong he Market in Zhoushan City, Zhejiang Province. The samples were processed according to the standard requirements. The head and shell of the shrimp were removed, and the edible portion was crushed and homogenized using a high-speed dispersion homogenizer and cryopreserved at −18 °C for subsequent use.

4-Hexylresorcinol standard (C_12_H_18_O_2_, purity 99%) was purchased from Alfa Aesar Company (Heysham, UK). Anhydrous sodium sulfate (analytical grade) was purchased from Sinopharm Chemical Reagent Co., Ltd. (Shanghai, China) Acetic acid (analytical grade) was purchased from Sigma (Saint Louis, MO, USA). Acetonitrile, methanol and ethyl acetate (all chromatographic purity) were purchased from Merck (Darmstadt, Germany); The water used in the experiment was ultrapure water prepared using Millipore-Q (Millipore, MA, USA).

### 3.2. Instruments and Equipment

ACQUITY UPLC XEVO TQ-S liquid chromatography-tandem mass spectrometer (with electrospray ion source) was from Waters Corporation (Milford, MA, USA). MS2 vortex mixer was from IKA (Staufen, Germany). Centrifuge 5810 high-speed centrifuge was from Eppendorf Corporation (Hamburg, Germany). R-201 rotary evaporator was from Shanghai Kexing Instrument Co., Ltd. (Shanghai, China); N-EVAP 112 nitrogen blowing instrument was from Organomation Company (Berlin, MA, USA); 12-channel solid-phase extraction device was from Supelco company (Bellefonte, PA, USA); Ultrasonic Cleaner was from Shanghai Kedao ultrasound Instrument Co., Ltd. (Shanghai, China); Organic microporous membrane (0.22 μm) was from Tianjin Jinteng test equipment Co., Ltd. (Tianjin, China); AL 204 electronic balance with sensitivity of 0.1 mg, SL-502N desktop balance with sensitivity of 0.01 g; Amino solid phase extraction column (60 mg/3 mL), C18 solid phase extraction column (1 g/6 mL), MAX (60 mg/3 mL), HLB (200 mg/6 mL) and PRIME HLB SPE solid phase extraction column (60 mg/3 mL) were from Waters Corporation (Milford, MA, USA).

### 3.3. Methods

#### 3.3.1. Solution Preparation

4-HR standard solution: the 100 mg/kg 4-HR standard stock solution was prepared by weighing 5.00 mg 4-HR standard product and placing it into a 50 mL volumetric flask. The 4-HR was dissolved in methanol and additional methanol was added up to the mark on the flask. The standard solution was sealed and stored at −20 °C in the refrigerator. The solution is stable for one month at −20 °C. The intermediate solution and the working solution were diluted to the desired mass concentration using organic solvent acetonitrile.

#### 3.3.2. Sample Extraction

A 5.00 g sample was weighed and placed into a 50 mL centrifuge tube with plug. Then, 10 g anhydrous sodium sulfate powder was added, and a further 10 mL methanol was added for extraction. The tube was capped and vortexed for 2 min and then extracted by ultrasound for 10 min. The extract was cooled to room temperature (25 °C) and centrifuged at 7000 rpm for 5 min. The supernatant was transferred to another 50 mL centrifuge tube, and 10 mL methanol was added to the residue. The extraction was repeated once, and all the extracts were combined, mixed homogeneously, and filtered with a quantitative filter paper (grade 52, Whatman, Hangzhou, China). After filtration, pure water was added to the 5 mL extract up to 10 mL. Following this step, the solution was ready for purification.

#### 3.3.3. Purification by Solid Phase Extraction Column

The Prime HLB SPE column was taken out and activated with 5 mL methanol and 5 mL water. Ten milliliters of the extract obtained in Section 3.3.2 was injected into the column. After loading the sample, 6 mL of 20% methanol water solution was used to rinse the column and the residual liquid from the solid phase column was squeezed until the column was dry. All of the above eluates were discarded. The target substance was eluted with 4 mL of 1% aqueous ammonia-methanol/acetonitrile (9:1, *v*/*v*) solution and the eluate was collected. The flow rate was less than 1 mL/min during the entire solid phase extraction process. The eluate was blown by nitrogen to near dry in a water bath at 40 °C. 1 mL 60% acetonitrile in water was added to dissolve the residue, and the mixture was filtered through a 0.22 μm organic microporous filter membrane for UPLC-MS/MS measurement.

#### 3.3.4. Conditions of UPLC

Waters Acquity UPLC BEH C18 (2.1 mm × 50 mm, 1.7 μm): injection volume: 5 μL; sample chamber temperature: 10 °C; column temperature: 40 °C; mobile phase A: acetonitrile; mobile phase B: water. The gradient elution program of the mobile phaseis shown in Table 2.

#### 3.3.5. Conditions for MS

Electrospray ion source: negative ion scanning mode; acquisition mode: multiple reaction monitoring (MRM); capillary voltage: 3.10 kV; desolvation gas: high purity nitrogen with flow rate of 800 L/h and temperature of 450 °C; cone gas: high purity nitrogen with flow rate of 50 L/h; collision gas: argon; source temperature: 150 °C. The MS parameters are shown in Table 3.

## 4. Conclusions

In this study, the UPLC-MS/MS method for the determination of 4-HR residues in shrimp was established by studying conditions required for UPLC-MS/MS and evaluating solid phase extraction columns and extraction conditions. We found that PRIME HLB solid phase extraction columns were effective for purification, especially when combined with the high sensitivity and strong separation effect of an ultra-high performance liquid chromatography-tandem mass spectrometer. Under these conditions, the experimental operation was simple and the matrix effect was low, thus increasing the recovery of the samples. The detection limit and the quantitation limit of this method were as low as 0.25 μg/kg and 0.80 μg/kg respectively, with good reproducibility and high precision, which enabled trace analysis of 4-HR in shrimp samples. Compared with previous studies, a confirmatory LC-MS/MS method was established with simple pretreatment, high sensitivity and wide applicability. This method is appropriate for 4-HR quantitation and can be used to replace routine LC-FLD analysis to maintain the safety of shrimp products containing 4-HR residues.

## Figures and Tables

**Table 1:** Recoveries and precision (RSDs) of 4-HR from spiked shrimp samples.

Samples	Spiked (μg/kg)	Mean Recovery/%	Intraday RSD (*n* = 6)/%	Interday RSD (*n* = 5)/%
Parapenaeopsis hardwichii	0.8	83.36	4.88	5.43
	4.0	84.01	5.13	4.82
	8.0	85.41	6.10	5.95
Fenneropenaeus chinensis	0.8	82.53	5.29	6.86
	4.0	88.23	3.57	5.04
	8.0	83.25	5.14	5.35
Palaemon gravieri	0.8	82.46	6.34	6.13
	4.0	85.83	4.31	5.53
	8.0	90.62	5.57	6.72
Acetes chinensis	0.8	81.35	5.36	5.76
	4.0	86.82	3.79	6.29
	8.0	82.33	4.66	6.51
Exopalaemon annadalei	0.8	81.62	6.32	6.87
	4.0	94.68	5.26	5.73
	8.0	85.19	5.81	5.54

**Table 2:** Gradient elution program of the mobile phase.

Time/min	Flow Rate/mL·min^−1^	Water/%	Acetonitrile/%	Gradient Curve
0.00	0.3	90.0	10.0	Initial
0.50	0.3	90.0	10.0	6
1.50	0.3	10.0	90.0	6
4.00	0.3	10.0	90.0	6
4.50	0.3	90.0	10.0	6
5.00	0.3	90.0	10.0	6

**Table 3:** Conditions of multiple reaction monitoring for 4-HR.

Compound Name	Parent (*m*/*z*)	Daughter (*m*/*z*)	Capillary Pressure/kV	Cone Pressure/V	Collision Energy/eV
4-HR quantitative ion pair	193.2	151.1	2.7	30	17
4-HR qualitative ion pair	193.2	122.0	2.7	30	20

## References

[1] Oujifard A., Seyfabadi J., Kenari A.A., Rezaei M. (2012). Growth and apparent digestibility of nutrients, fatty acids and amino acids in pacific white shrimp, litopenaeus vannamei, fed diets with rice protein concentrate as total and partial replacement of fish meal. Aquaculture.

[2] Ouraji H., Abedian Kenari A.M., Shabanpour B., Shabani A., Sodagar M., Jafarpour S.A., Ebrahimi G.H. (2011). Growth, survival, and fatty acid composition of indian white shrimp fenneropenaeus indicus (milne edwards) fed diets containing different levels of vitamin e and lipid. Aquacult. Int..

[3] Rosa R., Nunes M.L. (2004). Nutritional quality of red shrimp, aristeusantennatus (risso), pink shrimp, parapenaeus longirostris (lucas), and norway lobster, nephrops norvegicus (Linnaeus). J. Sci. Food Agric..

[4] Ayisi C.L., Hua X.M., Apraku A., Afriyie G., Beatrice Kyei B.A. (2017). Recent Studies Toward the Development of Practical Diets for Shrimp and Their Nutritional Requirements. HAYATI J. Biosci..

[5] Benjakul S., Visessanguan W., Tanaka M. (2005). Properties of phenoloxidase isolated from the cephalothorex of kuruma prawn (*Penaeus japonicus*). J. Food Biochem..

[6] Montero P., Martínez-Álvarez O., Zamorano J.P., Alique R., Gómez-Guillén M.C. (2006). Melanosis inhibition and 4-hexylresorcinol residual levels in deepwater pink shrimp (Parapenaeus longirostris) following various treatments. Eur. Food Res. Technol..

[7] Mcevily A.J., Radha I., Akiva G. (1991). Compositions and Methods for Inhibiting Browning in Foods Using Resorcinol Derivatives. U.S. Patent.

[8] Frankos V.H., Schmitt D.F., Haws L.C., Mcevily A.J., Iyengar R., Miller S.A., Munro L.C., Clydesdale F.M., Forbes A.L., Sauer R.M. (1991). Generally recognized as safe (GRAS) evaluation of 4-hexylresorcinol for use as a processing aid for prevention of melanosis in shrimp. Regul. Toxicol. Pharmacol..

[9] Iyengar R., Bohmont C.W., Mcevily A.J. (1991). 4 -hexylresorcinol and prevention of shrimp blackspot: Residual analyses. J. Food Comp. Anal..

[10] Alvarezparrilla E., Ladela R., Rodrigogarcia J., Escobedo-Gonzalez R., Mercado-Mercado G., Moyers-Montoya E., Vázquez-Flores A., González-Aguilar G.A. (2007). Dual effect of β-cyclodextrin (β-CD) on the inhibition of apple polyphenol oxidase by 4-hexylresorcinol (HR) and methyl jasmonate (MJ). Food Chem..

[11] Qiu F.Y., Ding L., Chen X.M. (2016). UPLC-MS/MS determination of 4-hexylresorcinol in Coprinus comatus. Phys. Test. Chem. Anal..

[12] Wang Y. (2015). Determination of 4-Hexylresorcinol in fresh pleurotus eryngii by ultra performance liquid chromatography tandem mass spectrometry. Chin. J. Anal. Lab..

[13] LóPezcaballero M.E., MartíNezáLvarez O., GóMezguilléN M.C., Montero P. (2006). Quality of Norway lobster (Nephrops norwegicus) treated with a 4-hexylresorcinol-based formulation. Eur. Food Res. Technol..

[14] Dai X.Y., Zhang M.X., Wei X.Y., Hider R.C., Zhou T. (2016). Novel multifunctional hydroxypyridinone derivatives as potential shrimp preservatives. Food Bioprocess Technol..

[15] Ying Y.U. (2015). Determination of 4-hexylresorcinol residue in crustacea by gas chromatography-mass spectrometry. J. Fujian Fish..

[16] Shokry R.F., Bebawy L.I., Elghobashy M.R., Abbas S.S. (2017). Comparative stability-indicating chromatographic methods for determination of 4-hexylresorcinol in pharmaceutical formulation and shrimps. J. Pharm. Biomed..

[17] Nakazato M., Matsumoto H., Kasuya Y., Yasuda K. (2005). Determination of 4-hexylresorcinol residues in prawns and crabs. Shokuhin seigaku zasshi. J. Food Hyg. Soc. Jpn..

[18] SelçUk A., Özden Ö. (2014). A rapid hplc method for determination of 4-hexylresorcinol residues in shrimp. J. Fish. Sci. Com..

[19] Yang W.G., Ha J.H., Kim S.G., Chae W.S. (2016). Spectroscopic determination of alkyl resorcinol concentration in hydroxyapatite composite. J. Anal. Sci. Technol..

[20] Jonker K.M., Dekker C.P. (2000). Determination of 4-hexylresorcinol in shrimp by liquid chromatography with fluorescence detection. J. Aoac. Int..

[21] Kim Y.H., Kim J.M., Lee J.S., Gang S.R., Lim H.S., Kim M., Lee O.H. (2016). Development and validation of an analytical method for the determination of 4-hexylresorcinol in food. Food Chem..

[22] Tang L., Deng Q., Xie J. (2015). Determination of residues of 4-hexylresorcinol in shrimp and crab by UPLC-MS/MS. Chin. Meas. Test..

[23] Li X.Q., Chao J., Sun Y.Y., Yang M.L., Chu X.G. (2009). Analysis of synthetic antioxidants and preservatives in edible vegetable oil by HPLC/TOF-MS. Food Chem..

[24] Eeckhaut A.V., Lanckmans K., Sarre S., Smolders I., Michotte Y. (2009). Validation of bioanalytical LC-MS/MS assays: Evaluation of matrix effects. J. Chromatogr. B.

[25] Wang Y., Liu X., Xiao C., Wang Z., Wang J., Xiao H., Cui L., Xiang Q., Yue T. (2012). HPLC determination of aflatoxin M1, in liquid milk and milk powder using solid phase extraction on OASIS HLB. Food Control.

